# EID1 promotes the response to canopy shade in *Arabidopsis thaliana* by repressing the action of phytochrome A

**DOI:** 10.17912/micropub.biology.001015

**Published:** 2023-12-12

**Authors:** Anne-Marie Staudt, Thomas Kretsch, Andreas Hiltbrunner

**Affiliations:** 1 Institute of Biology II, University of Freiburg, Germany; 2 Signalling Research Centres BIOSS and CIBSS, University of Freiburg

## Abstract

The phytochrome (phy) system enables plants to adapt to canopy shade. By sensing the reduction of the red:far-red light ratio in shade, phyA and phyB trigger downstream signalling cascades which eventually lead to enhanced elongation growth. In this study, we show that the F-box protein EID1 takes on an essential function within the shade avoidance response in *Arabidopsis thaliana* by repressing phyA action and thereby allowing seedlings to elongate in shade. Thus, altering EID1 activity provides a means to adapt the shade response without affecting phyB action and could have played a role in the evolution of shade tolerance.

**Figure 1. EID1 promotes shade-induced hypocotyl growth:**
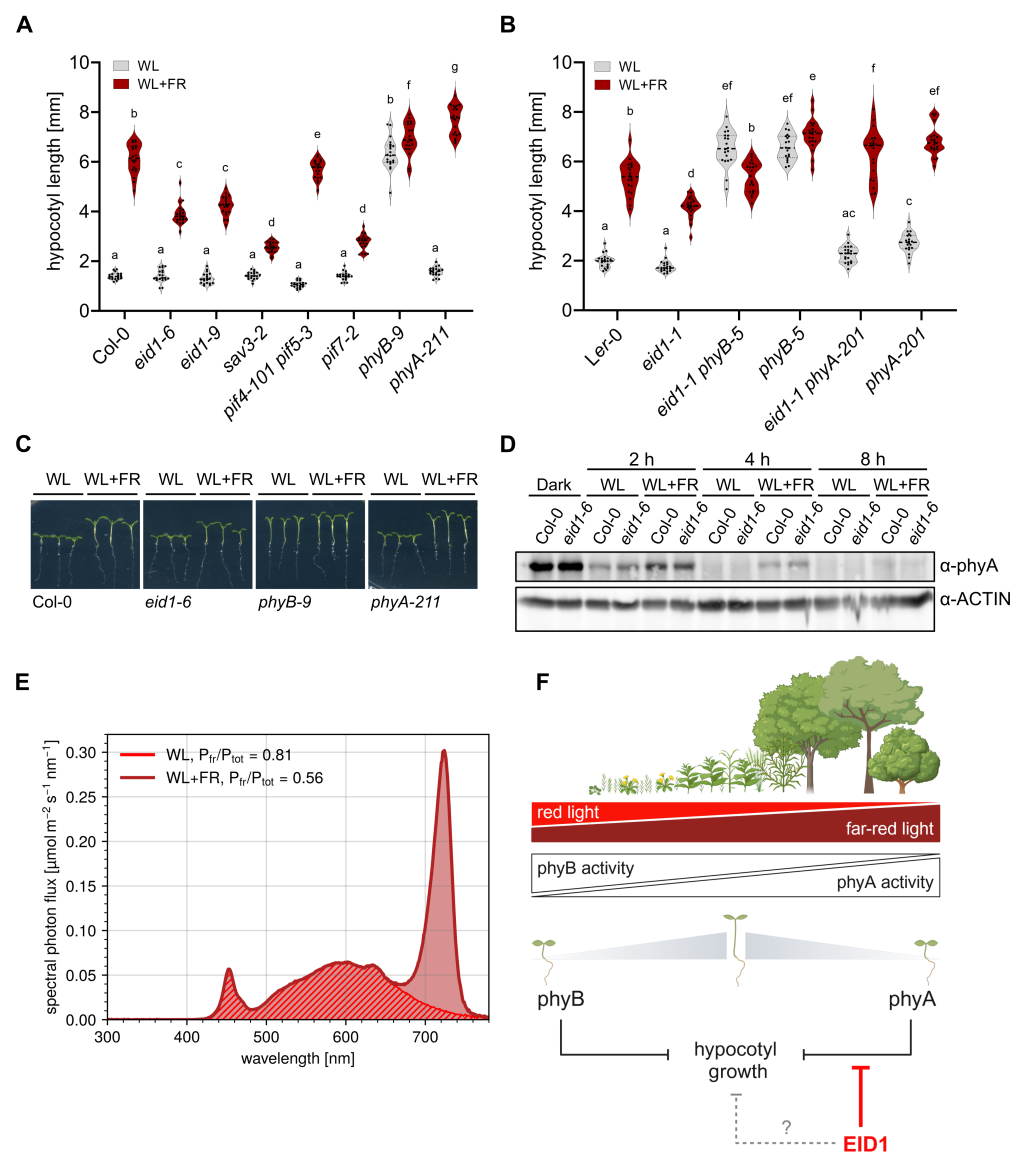
**(A-C)** Shade avoidance phenotype of 7-day-old seedlings. Seedlings of different genotypes were grown for 3 days in WL (35 µmol·m ^-2^ ·s ^-1^ ) at 21 °C and then transferred to either simulated shade (WL, 35 µmol·m ^-2^ ·s ^-1^ + FR, 35 µmol·m ^-2^ ·s ^-1^ ) or kept in WL for another 4 days. n=20. Letters indicate statistical significance determined by two-way ANOVA followed by post-hoc Tukeys HSD test; p<0.05. One representative experiment out of three replicate experiments is shown. **(D)** phyA protein levels. Col-0 and *eid1-6* seedlings were grown for 4 days in darkness and then transferred for 2 h, 4 h, and 8 h to either WL (35 µmol·m ^-2^ ·s ^-1^ ) or simulated shade (WL, 35 µmol·m ^-2^ ·s ^-1^ + FR, 35 µmol·m ^-2^ ·s ^-1^ ). Total protein extracts were analysed by SDS-PAGE and immunoblotting. phyA was detected in protein extracts using anti-phyA antibody. ACTIN1 served as loading control and was detected with α-Actin antibody. **(E)** Light spectra of the used WL and simulated shade (WL+FR) conditions. Relative levels of phytochrome in the active Pfr state (Pfr/Ptot) for the different light conditions were calculated according to Mancinelli (1994). **(F)** Working model of how EID1 modulates the shade avoidance response in *A. thaliana* . EID1 is repressing phyA activity, thereby releasing the repression of hypocotyl elongation. Published data and data presented in this study suggest that EID1 is largely specific for phyA, but minor phyA-independent effects cannot be ruled out at present. Figure was created with BioRender.com.

## Description

Limited light availability can threaten plant survival in high plant density environments and reduce yield in plantings of agronomically important crops. Neighbouring plants absorb large parts of the photosynthetically active radiation (PAR), including red (R) light, whereas far-red (FR) light is poorly absorbed and in part also reflected. Hence, compared to immediate sunlight, the red:far-red light ratio (R:FR) is strongly reduced in canopy shade or proximity of neighbouring plants. This reduction is sensed by R and FR light photoconvertible photoreceptors, named phytochromes (phys). PhyA and phyB are conserved in seed plants and it is well established that phyB is activated by light with a high R and low FR content, while phyA is most active in light with a high FR content. Both phyA and phyB suppress hypocotyl elongation in seedlings when activated under the respective light condition (Legris et al., 2019) .

In shade-intolerant plants, including the model plant *Arabidopsis thaliana* and many important crops, low R:FR initiates a set of adaptive responses, collectively known as the shade avoidance response (SAR). SAR aims at outgrowing plants in close proximity, thereby optimising photosynthetic activity by increasing growth at the expense of crop yield and defence. PhyB is believed to be the main regulator of SAR, repressing this response in sunlight but not in shade (Franklin et al., 2008; Casal et al., 2012) . Function of phyA in SAR was shown to be most important during de-etiolation and inhibition of hypocotyl elongation under deep shade conditions, antagonising the effect of phyB (Yanovsky et al., 1995; Martinez-Garcia et al., 2014) . In contrast to shade-intolerant plants, shade-tolerant species do not increase growth in shade conditions. Recent findings show that a highly efficient phyA-dependent pathway supresses the SAR in the shade-tolerant species *Cardamine hirsuta* , a close relative of Arabidopsis (Molina-Contreras et al., 2019) . This suggests that in Arabidopsis and potentially other shade-intolerant species, this phyA-dependent pathway could be inactivated, leading to the induction of SAR by inactivation of phyB in low R:FR conditions.

EMPFINDLICHER IM DUNKELROTEN LICHT 1 ( EID1 ) is one of the very few light signalling components known that specifically affect phyA action, making EID1 an interesting candidate to examine in the context of SAR (Büche et al., 2000). It was postulated that EID1 is an F-box protein, which as part of an SCF ubiquitin-ligase complex targets positive components of the phyA signalling cascade for degradation via the proteasome (Dieterle et al., 2001; Marrocco et al., 2006) .

It was already shown that adult *eid1* mutant plants exhibit reduced petiole elongation in simulated shade (Marrocco et al., 2006) . In the presented work, we examined whether non-functional EID1 also leads to alterations in shade induced hypocotyl elongation at seedling stage. Indeed, *eid1* mutants in the Col-0 as well as in the L *er* -0 background show a clear hyposensitive response to simulated shade (WL + FR) with reduced hypocotyl growth compared to the wildtype. This suggests that EID1 -dependent repression of phyA action is required for full induction of the SAR ( Figure 1A -C). In order to assess the importance of EID1 for SAR, well-described mutants exhibiting a strong reduction in SAR were used as reference. The included mutants are on one hand *sav3-2* , which is impaired in biosynthesis of the growth-promoting phytohormone auxin, and on the other hand the *pif4-101 pif5-3* double and *pif7-2* single mutant, which are lacking essential components of phy signalling pathways (Tao et al., 2008; Lorrain et al., 2008; Koini et al., 2009; Li et al., 2012) . Although *sav3-2* and *pif7-2* show an even more pronounced phenotype in simulated shade than *eid1-6* and *eid1-9* seedlings, EID1 deficient seedlings clearly show a stronger impairment in SAR than the *pif4-101 pif5-3* mutant. EID1 therefore plays an essential role in SAR.

The observation that both *phyA-201* single and *eid1-1 phyA-201* double mutants showed a hypersensitive response in simulated shade confirms that EID1 acts via phyA-dependent mechanisms within the SAR. On the other hand, *eid1-1 phyB-5* seedlings showed an intermediate phenotype compared to *eid1-1* and *phyB-5* single mutants in shade. This finding is in good agreement with the assumption that phyA-dependent repression of hypocotyl elongation is still intact in the *phyB-5* mutant and enhanced through the absence of EID1 .

How EID1 is functioning on a molecular level is still not understood. As EID1 is an F-box protein, it stands to reason that EID1 induces phyA protein degradation in shade. Büche et al. (2000) already analysed phyA degradation rates in seedlings lacking functional EID1 under different light conditions. Their findings strongly suggest that EID1 is not involved in the degradation of phyA in monochromatic light. It is however possible that phyA degradation is dependent on EID1 under specific, more natural light conditions, like for example in canopy shade. To test for possible alterations of phyA levels and degradation kinetics in shade conditions, Col-0 and *eid1-6* seedlings were grown for 4 days in darkness and then transferred for 2 h, 4 h, and 8 h to either simulated shade or WL. phyA protein levels were analysed via immunoblotting ( Figure 1D ). At any time-point tested, phyA protein levels were higher in simulated shade when compared to WL. These findings suggest that phyA is stabilised by simulated shade, which is in good agreement with results from Martinez-Garcia et al. (2014). However, the *eid1-6* mutation did not have any detectable effect on phyA stability in WL nor in simulated shade, showing that even in shade conditions, EID1 is unlikely to control phyA action by targeting phyA for degradation.

Although a potential effect of EID1 through other factors has not yet been tested in detail, previous reports together with our findings suggest that EID1 is largely specific for phyA (Büche et al., 2000; Dieterle et al., 2001). Our data strongly suggest that EID1 suppresses the negative effect of phyA on SAR, making it an essential component for the response to shade conditions in Arabidopsis and possibly other shade-intolerant species. Thus, altering EID1 activity would allow plants to fine-tune the response to shade conditions without interfering with the general role of phyB in regulation of growth and development ( Figure 1E, F ). This makes *EID1* a potential target in the evolution of shade-tolerance, since weak *EID1* alleles or loss-of-function *EID1* alleles would lead to increased phyA activity and thereby suppress shade-induced growth. Apart from EID1 , only few phyA-specific factors have been identified so far, including FHY1/FHL, LAF1, LAF3, LAF6, and NOT9B (Whitelam et al., 1993; Zhou et al., 2005; Ballesteros et al., 2001; Hare et al., 2003; Møller et al., 2001; Schwenk et al., 2021). However, their effect on SAR has not yet been investigated in detail.

In this study, we showed that seedlings deficient in EID1 are unable to suppress the negative effect of phyA on hypocotyl growth in shade conditions, establishing EID1 as important factor in the SAR. The molecular mechanism underlying the effect of EID1 on phyA-dependent repression of the SAR is still unknown, but it is unlikely that EID1 acts by promoting the degradation of phyA in shade conditions.

## Methods

**Light conditions**

Seedlings were grown at 21 °C in HettCube 400R incubators (Hettich Lab Technology, Beverly, USA) equipped with LED lights of different light quality. For white light conditions (WL) a fluence rate of 35 µmol·m ^-2^ ·s ^-1^ was chosen. For simulated shade conditions (WL+FR) 35 µmol·m ^-2^ ·s ^-1^ of white light were supplemented with 35 µmol·m ^-2^ ·s ^-1^ of far-red light (λ = 740 nm). White and far-red light fluence rates were measured using a LI-250 light meter equipped with a LI-190R Quantum sensor (LI-COR, Bad Homburg, Germany) and a X1 Optometer equipped with a PS-3703-4 sensor (Gigahertz-Optik, Türkenfeld, Germany) respectively. Light spectra of conditions used in all experiments were measured using a AvaSpec-ULS2048-USB2-FCPC spectrometer (Avantes B.V., Apeldoorn, Netherlands) and are shown in Figure 1E . Pfr/Ptot levels were calculated according to Mancinelli (1994). We also calculated the R:FR ratio (Extended Data), which differs depending on the R and FR wavelength ranges used for the calculation. The numbers given in the plots in the Extended Data are based on the definition by Whitelam and Franklin (2005) (R = 655-665 nm; FR = 725-735 nm) and Martinez-Garcia et al. (2014) (R = 640-670 nm; FR = 720-750 nm); it should be noted that it is not possible to conclude on the Pfr/Ptot level based on the R:FR ratio alone, since wavelength ranges that are not taken into account in the R:FR ratio also influence the photoconversion of phytochromes.

**Phenotypic analysis**

*Arabidopsis thaliana* (L.) Heynh. wildtype and mutant seeds (see Reagents) were sown on 1/2 MS media (0.98 g·l ^-1^ MES, 2.15 g·l ^-1^ MS salts, 10 g·l ^-1^ Bacto Agar, pH 5.7) and stratified for 3 days in darkness at 4 °C. Each plate was prepared as duplicate. After stratification, all plates were transferred to WL conditions. After 3 days of growth, one plate of each duplicate was transferred to simulated shade (WL+FR). Seedlings were grown for another 4 days. 20 seedlings per genotype and light conditions were fixed and scanned for subsequent hypocotyl measurements using ImageJ software.

**Protein level analysis**

Col-0 and *eid1-6* seeds were sown on 1/2 MS media and stratified for 3 days in darkness at 4 °C. Germination was induced for 6 h in WL at 21 °C. Then, the seedlings were grown for 4 days in darkness at 21 °C before transfer to either WL or WL+FR conditions for different amounts of time. Proteins were extracted from the seedlings under denaturing conditions (65 mM Tris/HCl pH 6.8, 4 M urea, 10 % glycerol, 3 % SDS, 0.05 % bromophenol blue). Total protein concentration of the extracts was measured using the amido black method (Popov et al., 1975) . 20 µg of total protein extracts were separated by 10 % sodium dodecyl sulphate-polyacrylamide gel electrophoresis. The gel was then blotted on Immobilon-P PVDF membrane (Merck Millipore, Billerica, USA) using the Wet Blotting system (Bio-Rad Laboratories, Hercules, USA). Actin was detected by the anti-Actin antibody at 1:10000 dilution (10-B3; Sigma-Aldrich, Saint Louis, USA). phyA was detected using the anti-phyA antibody at 1:2000 dilution (AS07 220; Agrisera AB, Vännäs, Sweden).

## Reagents

**Chemicals**

**Table:** 

**Chemical**	**Supplier**
2-(N-morpholino)ethanesulfonic acid (MES)	Carl Roth (Karlsruhe, Germany), 4256.2
Bacto Agar	Becton, Dickinson and Company (Franklin Lakes, USA), 214010
Murashige & Skoog medium (MS salts)	Duchefa Biochemie (Haarlem, Netherlands), M0221

**Gene accession numbers**

**Table:** 

**Gene**	**Accession number**
*EID1*	AT4G02440
*PHYA*	AT1G09570
*PHYB*	AT2G18790
*TAA1*	AT1G70560
*PIF4*	AT2G43010
*PIF5*	AT3G59060
*PIF7*	AT5G61270

**Plant lines used in this study**

**Table:** 

**Plant line**	**Background**	**Reference**
Col-0		
L *er* -0		
*eid1-6*	Col-7	Marrocco et al. (2006)
*eid1-9*	Col-0	SALK_027403, Alonso et al. (2003)
*sav3-2* (a *taa1* mutant allele)	Col-0	Tao et al. (2008)
*pif4-101 pif5-3*	Col-0	De Lucas et al. (2008)
*pif7-2*	Col-0	Leivar et al. (2008)
*phyB-9*	Col-0	Reed et al. (1993)
*phyA-211*	Col-0	Reed et al. (1994)
*eid1-1*	L *er* -0	Dieterle et al. (2001)
*eid1-1 phyB-5*	L *er* -0	Büche et al. (2000)
*phyB-5*	L *er* -0	Reed et al. (1993)
*eid1-1 phyA-201*	L *er* -0	this study
*phyA-201*	L *er* -0	Reed et al. (1994)

## Extended Data

Description: For the light spectra shown in Figure 1E (white light, WL; simulated shade, WL+FR), the R:FR ratio was calculated according to Whitelam and Franklin (2005) (R = 655-665 nm, FR = 725-735 nm) or Martinez-Garcia et al. (2014) (R = 640-670 nm, FR = 720-750 nm). The R and FR wavelength ranges taken into account for the calculations are indicated in the plots.. Resource Type: Image. DOI: 10.22002/4sscy-r7569
